# Rituximab in patients with acute ST-elevation myocardial infarction: an experimental medicine safety study

**DOI:** 10.1093/cvr/cvab113

**Published:** 2021-03-30

**Authors:** Tian X Zhao, Muhammad Aetesam-Ur-Rahman, Andrew P Sage, Saji Victor, Rincy Kurian, Sarah Fielding, Hafid Ait-Oufella, Yi-Da Chiu, Christoph J Binder, Mikel Mckie, Stephen P Hoole, Ziad Mallat

**Affiliations:** 1 Division of Cardiovascular Medicine, Department of Medicine, University of Cambridge, Cambridge, UK; 2 Department of Cardiology, Royal Papworth Hospital NHS Foundation Trust, Cambridge, UK; 3 Research and Development, Royal Papworth Hospital NHS Foundation Trust, Cambridge, UK; 4 Papworth Trials Unit Collaboration, Royal Papworth Hospital, Cambridge, UK; 5 Université de Paris, Inserm U970, Paris-Cardiovascular Research Center, Paris, France; 6 Department of Laboratory Medicine, Medical University of Vienna, Vienna, Austria

**Keywords:** Myocardial infarction, B lymphocytes, Rituximab, Immune system

## Abstract

**Aims:**

In pre-clinical models of acute myocardial infarction (MI), mature B cells mobilize inflammatory monocytes into the heart, leading to increased infarct size and deterioration of cardiac function, whilst anti-CD20 antibody-mediated depletion of B cells limits myocardial injury and improves cardiac function. Rituximab is a monoclonal anti-CD20 antibody targeted against human B cells. However, its use in cardiovascular disease is untested and is currently contraindicated. Therefore, we assessed the safety, feasibility, and pharmacodynamic effect of rituximab given to patients with acute ST-elevation MI (STEMI).

**Methods and results:**

Rituximab in patients with acute ST-elevation myocardial infarction (RITA-MI) was a prospective, open-label, dose-escalation, single-arm, phase 1/2a clinical trial, which tested rituximab administered as a single intravenous dose in patients with STEMI within 48 h of symptom onset. Four escalating doses (200, 500, 700, and 1000 mg) were used. The primary endpoint was safety, whilst secondary endpoints were changes in circulating immune cell subsets including B cells, and cardiac and inflammatory biomarkers. A total of 24 patients were dosed. Rituximab appeared well tolerated. Seven serious adverse events were reported, none of which were assessed as being related to the rituximab infusion. Rituximab caused a mean 96.3% (95% confidence interval 93.8–98.8%) depletion of circulating B cells within 30 min of starting the infusion. Maximal B-cell depletion was seen at Day 6, which was significantly lower than baseline for all doses (*P* < 0.001). B-cell repopulation at 6 months was dose-dependent, with modulation of returning B-cell subsets. Immunoglobulin (IgG, IgM, and IgA) levels were not affected during the 6 months of follow-up.

**Conclusions:**

A single infusion of rituximab appears safe when given in the acute STEMI setting and substantially alters circulating B-cell subsets. We provide important new insight into the feasibility and pharmacodynamics of rituximab in acute STEMI, which will inform further clinical translation of this potential therapy.

**Clinical trial registration:**

NCT03072199 at https://www.clinicaltrials.gov/

## Graphical Abstract

**Figure:**
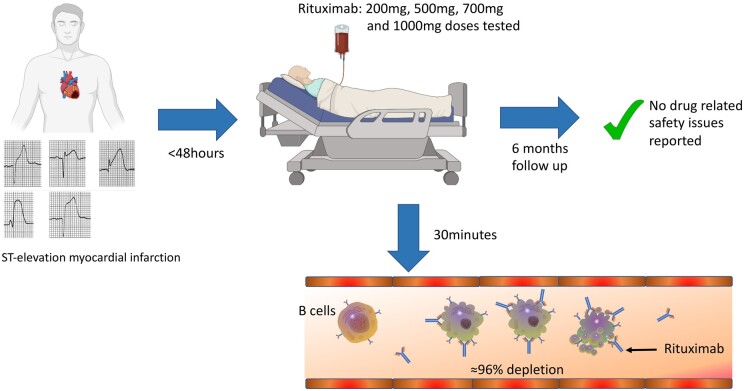

## 1. Introduction

Survival after acute myocardial infarction (MI) has improved over the past decades due to advances in medical therapy and the use of percutaneous coronary intervention (PCI). However, despite multiple clinical trials, medical therapy to reduce infarct size and assist in ischaemic cardiac remodelling remains limited.^1^^,^^2^ As a result, the global prevalence of ischaemic heart failure is still rising, resulting in an increasing global burden of disease.^3^ Despite significant advances in available therapies, mortality and morbidity for heart failure remain high and quality of life may be limited.^4^

Immediately after an MI, the innate immune response is activated and results in infiltration of neutrophils and monocytes into the infarcted myocardium. Detailed study of the role of neutrophils and monocytes have shown varied functions depending on both timing and subset. For example, it appears that neutrophils can worsen cardiac function after MI due to an excessive inflammatory response but also subsequently increase macrophage efferocytosis, which is likely benefical.^5^^,^^6^ Furthermore, it appears that mobilization of monocytes from bone marrow after MI occurs in a biphasic manner with initial inflammatory Ly-6C^high^ and then reparative Ly-6C^low^ monocytes.^7^ Focus has also been on the adaptive immune system where pre-clinical experimental work has shown that mature B cells are instrumental in the orchestration of the inflammatory response after ischaemic myocardial injury, in part through mobilization of inflammatory monocytes to the infarct site. Furthermore, B-cell depletion using a single injection of anti-CD20 antibody after MI led to a reduction in infarct size, end-systolic left ventricular (LV) dimension, and an increase of LV myocardial contractility.^8^ Similar cardio-protective effects have been described in association with reduced B-cell activation.^9^ In a linked paradigm, acute MI triggers an inflammatory process which has been shown to accelerate the progression of atherosclerotic plaques, and might clinically result in patients having an increased rate of further cardiovascular events in the intervening period.^10^ B cells appear to have an important role in atherosclerosis development. B-cell progenitor cells give rise to distinct B1- and B2-cell lineages which appear, in pre-clinical models, to have different roles in atherosclerosis. Innate B1 cells secrete high levels of IgM antibodies to oxidation-specific epitopes (OSEs) which are thought to be atheroprotective, whilst B2 cells give rise to both atherogenic adaptive follicular and germinal centre B cells, and atheroprotective innate-like marginal zone B cells.^11^^,^^12^ Pre-clinical evidence has shown that B-cell depletion using anti-CD20 antibody, which removes B2 cells but does not affect B1 cells in mice, reduces atherosclerotic lesion development^13^^,^^14^ and therefore may provide a double-hit therapeutic strategy post-MI. In approximately, 1000 patients admitted with acute MI, biomarkers of B-cell activation at admission were associated with a substantially higher risk of death and recurrent MI after 2 years of follow-up, even after adjustment for several multivariable risk factors.^8^

Rituximab is a monoclonal antibody targeted against human B cells and has been used in the treatment of certain autoimmune diseases and cancers.^15^ However, its use in cardiovascular disease is untested and is currently contraindicated due to a lack of evidence and limited reports of cardiovascular complications.^16^ We therefore carried out an early phase experimental medicine trial with the aim to assess the safety and feasibility of a single infusion of rituximab given acutely in patients with ST-elevation MI (STEMI), to determine its effect on B-cell counts acutely and on follow-up, and address its impact on a number of other biological and clinical exploratory endpoints.

## 2. Methods

### 2.1 Trial design

Rituximab in patients with acute ST-elevation myocardial infarction (RITA-MI) was an academically driven, prospective, single-centre, open-label, single-arm, dose-escalation, phase 1/2a clinical trial. It was performed at the Royal Papworth Hospital, Cambridge, UK with overall study co-ordination provided by the Papworth Trials Unit Collaboration. The study received a favourable opinion by the Essex Research Ethics Committee, UK (16/EE/0241). All study procedures were conducted after formal written consent, in accordance with the Declaration of Helsinki. Trial registration number: Clinicaltrials.gov (NCT03072199).

### 2.2 Study participants

This trial included patients aged between 18 and 75 years old who presented with acute STEMI with successful primary percutaneous coronary intervention (PPCI) during the first 24 h of cardiac chest pain onset. When the onset was staggered or crescendo, the timepoint of worst chest pain was used. Exclusion criteria included a previous history of STEMI; cardiogenic shock; cardiac electrical instability; severe congestive heart failure; residual severe proximal bystander disease awaiting inpatient revascularization; corrected QT interval >500 ms; haematologic abnormalities (haemoglobin <10 g/dL or haematocrit <30%, platelet cell count of <100 ×103/µL, white blood cell count <4 ×103/µL); hypogammaglobulinaemia (defined as IgG<3 g/L); renal failure (estimated glomerular filtration rate by the MDRD formula < 45 mL/min/1.73 m^2^); known hepatic failure or abnormal liver function tests at baseline (alanine aminotransferase (ALT) > 2× upper range of normal); active or recurrent hepatitis B virus; current or previous tuberculosis (based on history and chest X-ray); current infections (including history of HIV); presence or history in the previous 5 years of cancer (except *in situ* cancer of the cervix or basal cell carcinoma); any oral or intravenous immunosuppressive, imunomodulatory or immunodepleting treatment; expected need for vaccination with a live attenuated vaccine during the study; known or suspected pregnancy; lactating women; and women of childbearing age.

### 2.3 Recruitment

Patients were recruited after presenting on the PPCI pathway. This is a regional pathway for all patients identified as having a STEMI by the paramedics or referring regional hospitals. Patients were approached after transfer to the coronary care unit and after the effects of any narcotic had worn off.

### 2.4 Trial procedures

After informed consent, patients were screened for eligibility, including hepatitis B virus screening and a chest X-ray for any evidence of pulmonary tuberculosis. The next day, and within 48 h of worse symptom onset, the patient was given an infusion of premedication, which included an intravenous infusion of 100 mg of methylprednisolone over 30 min, an intravenous injection of 10 mg of chlorphenamine, and 1 g of oral paracetamol. Pre-medication was constant for all patients and routinely given with rituximab in other diseases to mitigate the common side effect of rituximab infusion-related reaction. Thirty minutes after pre-medication, an infusion of rituximab was started at a rate of 50 mg/h for the first 30 min, increasing in 50 mg/h increments every 30 min up to a maximum rate of 400 mg/h if tolerated. Four sequential escalating dose groups were used: 200, 500, 700, and 1000 mg. After the completion of the infusion, no further interventions were given, and patients were followed up daily whilst they were inpatients. Discharge was clinically led. After discharge, patients were seen as outpatients on days 6 and 14, and at 6 months. In addition, telephone follow-up was performed on day 30 and at 3 months.

### 2.5 Endpoints

The primary endpoint was safety, which was assessed through: (i) a review of adverse events (AEs) as classified by use of the Medical Dictionary for Regulatory Activities; (ii) review of concomitant medications; (iii) physical examination; (iv) changes in safety bloods (defined in Supplementary material online, *Methods*); (v) vital observations (defined as temperature, blood pressure, heart rate, oxygen saturation, and respiratory rate); (vi) electrocardiograms and continuous cardiac monitoring before, during and after the infusion. Secondary/exploratory outcomes included: (i) change in circulating B cells and their subsets measured by fluorescence-activated cell sorting (FACS) assessed at baseline, 30 min, 6 h, day 6, day 14, and 6 months post-infusion initiation; (ii) change in other immune cell counts by FACS and on clinical bloods; (iii) change in cardiac and inflammatory biomarkers including C-reactive protein (CRP), interleukin-6 (IL-6), troponin I (troponin was not measured at 6 months), and NT-pro-B-type natriuretic peptide (NT-pro-BNP) assessed at baseline, day 1/2, day 6, and 6 months post-infusion; and (iv) change in immunoglobulin counts and lipids. Details of FACS and biomarker analysis are found in the Supplementary material online, *Methods*.

All study data and particularly, detailed safety data, was reviewed by an independent Data Monitoring and Safety Board (DMSB) before progression to the subsequent dose group. At the end of the study, the DMSB reviewed all of the trial safety data and gave a written opinion on the safety outcomes.

### 2.6 Statistical analysis

Being a phase 1/2a dose ranging study, no sample size calculation was possible, the results obtained here will guide future sample size calculations for a phase 2b clinical study. A statistical plans was developed before study completion and the statistical analysis was undertaken by an independent biostatistician with access to all the data. For summary statistics, continuous variables are reported using means and standard deviation, and categorical variables using frequency and percentages. For the primary outcome, the frequency of AEs per patient was summarized for each event based on dose level. As the analysis is descriptive in nature hypothesis testing was not undertaken.

For the secondary outcomes, pre-specified statistical analysis was undertaken against the null hypotheses by two-way repeated measure analysis of variance (rANOVA) using both dose and study timepoint as independent variables. In cases where the rANOVA indicated a significant contribution from either variable or the interaction term, one-way ANOVA was used to look at either dose or timepoint individually and *post hoc* paired *t*-tests to investigate at which timepoint/dose comparisons were significantly different. A Bonferroni correction for multiple comparison, Shapiro–Wilk test for normality, and Levene’s test for homogenous variances was used. Statistical analyses were conducted using R version 4.0.1 software.

## 3. Results

**Figure 1:**
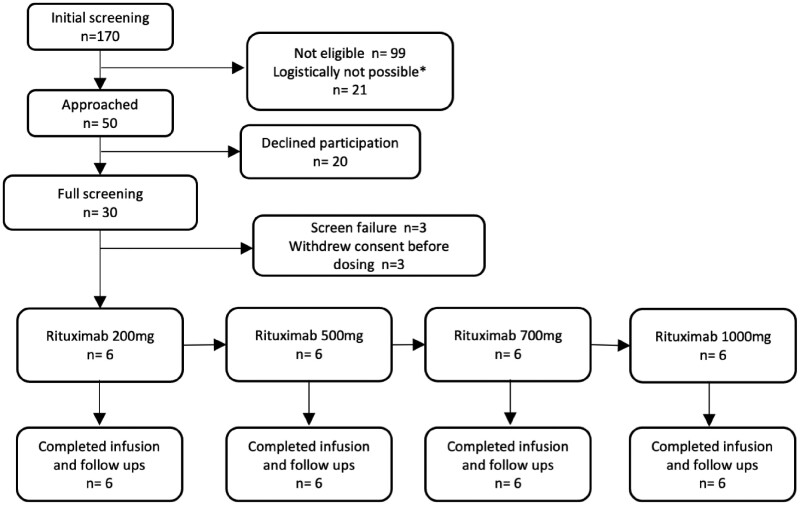
Consort diagram for RITA-MI. All patients completed dosing and follow-up. ^a^Logistically not possible = dosing would have been on weekend which was not permitted, doctor/nurse not available to dose patient, mandated follow-up would have been on weekend or doctor not available. Top three reasons for ineligibility were timing of chest pain, previous medical history, clinical instability post-MI.

Between September 2017 and February 2019, 170 patients underwent initial screening via medical notes at Royal Papworth Hospital, UK. Of these, 50 were eligible and approached for informed consent. Of the 50 patients, 20 declined to take part, and 30 patients were consented and went on to have full screening. There were three screen failures and a further three patients withdrew their consent before dosing. In total, 24 patients were dosed across four dose groups (200, 500, 700, and 1000 mg) with six patients in each group (*Figure 1*). For all patients, the mean time from symptom onset to starting the infusion was 43.1 h (SD 7.9 h) with no statistical difference between groups. All patients completed the infusion and all follow-ups. The baseline demographics are summarized in *Table 1*.

**Table 1:** Summary of demographics for RITA-MI

	Rituximab dose			
	200 mg (*n* = 6)	500 mg (*n* = 6)	700 mg (*n* = 6)	1000 mg (*n* = 6)
Age				
Mean (SD)	58.5 (10.4)	52.5 (9.5)	65 (8.5)	59.3 (8.0)
Median (min–max)	60.5 (40–70)	51.5 (38–66)	68 (49–72)	59 (51–73)
Sex				
Male	5 (83%)	6 (100%)	6 (100%)	5 (83%)
Race				
White or Caucasian	6 (100%)	6 (100%)	6 (100%)	6 (100%)
BMI				
Mean (SD)	28.7 (2.3)	26.8 (4.1)	29.5 (6.2)	28.1 (3.0)
Presentation				
PPCI	6 (100%)	6 (100%)	6 (100%)	6 (100%)
Previous medical history of:				
NSTEMI	0 (0%)	1 (17%)	0 (0%)	1 (17%)
PCI/CABG	0 (0%)	0/1 (17%)	0 (0%)	1 (17%)/0
Arrhythmias	0 (0%)	0 (0%)	1 (17%)^a^	0 (0%)
Hypercholesteraemia	0 (0%)	1 (17%)	4 (67%)	2 (33%)
Hypertension	3 (50%)	2 (33%)	3 (50%)	4 (67%)
Diabetes	0 (0%)	0 (0%)	1 (17%)	1 (17%)
Smoking				
Current/ex-smoker/never	2/1/3	2/1/3	1/3/2	2/3/1
Medication:				
Aspirin	6 (100%)	6 (100%)	6 (100%)	6 (100%)
Clopidogrel/ticagrelor	6 (100%)	6 (100%)	6 (100%)	6 (100%)
Statin	6 (100%)	6 (100%)	6 (100%)	6 (100%)
β-blocker	6 (100%)	5 (83%)	5 (83%)	5 (83%)
ACEi/ARB	5 (83%)	5 (83%)	5 (83%)	5 (83%)
Culprit lesion/dosing				
LAD	5 (83%)	4^b^ (67%)	1 (17%)	1 (17%)
LCx	0 (0%)	2 (33%)	2 (33%)	1 (17%)
RCA	1 (17%)	0 (0%)	3 (50%)	4 (67%)
Mean number of stents	1.5 (0.5)	1 (0)	1.5 (0.8)	2 (1.3)
Mean hours between onset of MI to start of dosing	44.8 (4.5)	39.3 (8.8)	43 (5.6)	45.5 (11.4)

Continuous variables are expressed using mean and standard deviation, whilst categorical variables are expressed using frequency and percentage.

ACEi, angiotensin-converting enzyme inhibitors; ARB, angiotensin II receptor blockers; BMI, body mass index; LAD, left anterior descending; LCx, left circumflex; PPCI, primary percutaneous coronary intervention; RCA, right coronary artery.

a Patient had atrial fibrillation.

b One of the lesions was an intermediate coronary artery.

### 3.1 Safety of rituximab

In total, there were 64 AEs in 20/24 patients balanced across the dose groups (*Table 2* with a full AE list in Supplementary material online, *Table S1*). There were seven serious AEs (SAEs) in total. Two were related to an elective admission for a staged PCI procedure for coronary stenosis in a non-culprit vessel seen during index PPCI and which was deferred at that time. Both procedures were already planned at the time of recruitment into the study. The other five SAEs were all assessed as unrelated to rituximab and are described in detail in Supplementary material online, *Table S2*. The most common groups of AEs were gastrointestinal disturbances, which included diarrhoea, indigestion, and abdominal pain. Chest pain was seen and was consistent with symptoms experienced in patients post-MI. None of the chest pain AEs occurred during or within 24 h of infusion and none were assessed as related to rituximab.

**Table 2:** Summary of adverse and serious adverse events (AEs) for RITA-MI

	Dose group			
	200 mg	500 mg	700 mg	1000 mg
Number of patients	6	6	6	6
Adverse events (AEs)				
Number (no. of patients)	18 (6)	19 (5)	10 (5)	17 (4)
Number definitely or probably related^a^	3	0	0	0
Number possibly related^a^	3	3	1	1
Number not related	12	16	9	16
Number of AEs graded				
Severe	0	1^b^	0	0
Moderate	1	7	3	2
Mild	17	11	7	15
Serious adverse events (SAEs)				
Elective procedures recorded as SAE^c^	1	0	0	1
Other SAEs	0	4	1	0
Number attributed to Rx	0	0	0	0
Common AEs				
GI disturbance^d^	5	5	2	4
Elevated LFTs	2	1	0	2
Chest pain	1	3	1	0
Infusion related reaction^e^	3	0	0	0
Infections	1	1	1	1

GI, gastrointestinal; LFTs, liver function tests; PCI, percutaneous coronary intervention; Rx, rituximab.

a AE are assessed to be either definitely, probably, possibly, or not related to rituximab infusion.

b Additionally reported as SAE. Event was ventricular tachycardia requiring admission- see text.

c For this trial, elective procedures are recorded as SAEs and reported separately here. Both SAEs are elective staged PCI on non-culprit vessels.

d GI disturbance include diarrhoea, indigestion, and abdominal pain.

e Infusion-related reactions are commonly seen during the intravenous admission of biologics and include flushing, headache, fever, and nausea.

Infusion-related reactions occurred in three patients. The infusion was not stopped in any of the patients. For two patients, the rate of infusion was reduced and the symptoms resolved.

Infections occurred in four patients, two were cold/flu during the flu season several months after rituximab infusion. One was a urinary tract infection 5 months after infusion and required antibiotic treatment. The remaining infection was a lower respiratory infection, which required antibiotics and occurred 1 week after discharge.

The independent Data Safety and Monitoring Board assessed all the safety data at end of the trial and found no concerning safety issues.

### 3.2 Effect of rituximab on circulating B cells

**Figure 2:**
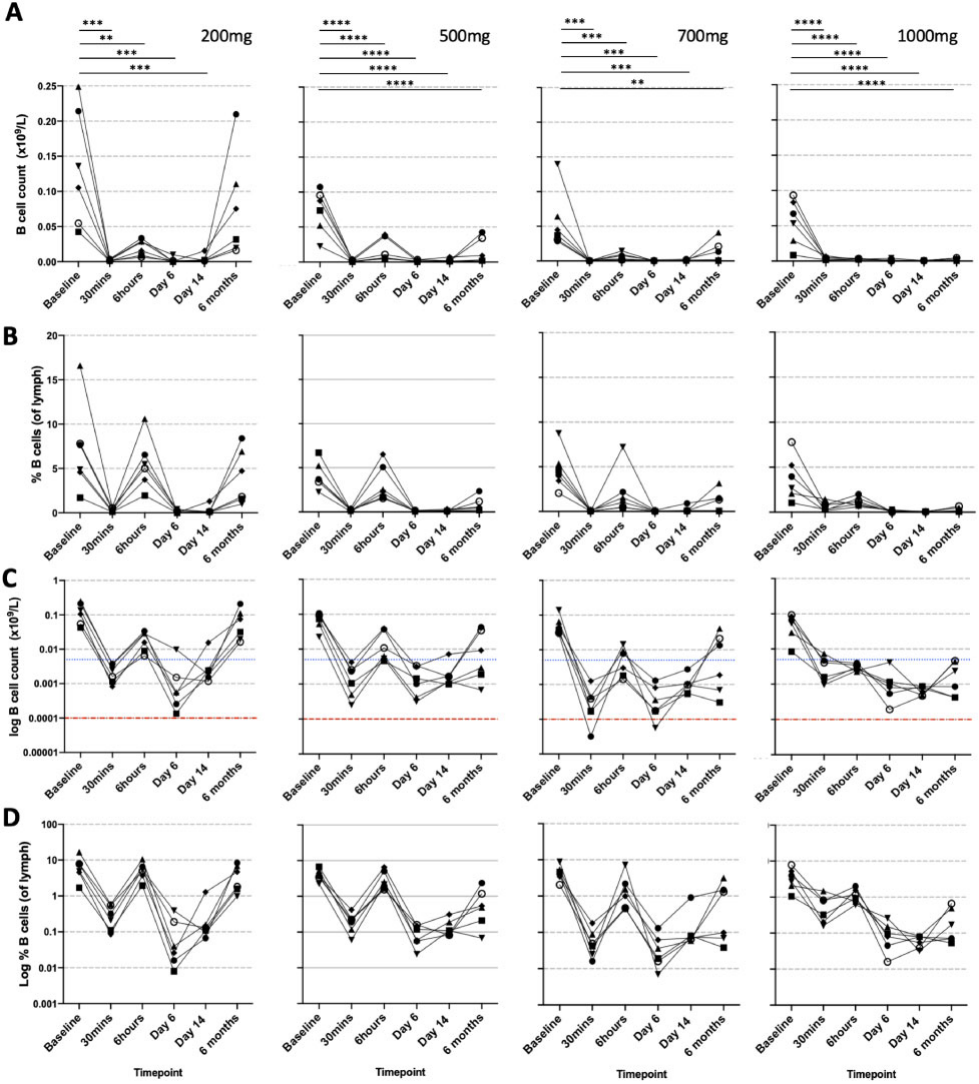
CD19+ B-cell depletion. Panel (*A* and *B*) shows the effect of four different doses of rituximab (200, 500, 700, and 1000 mg) on B-cell count and percentage (of lymphocytes) respectively. Each point and line represent a single patient in each group. Panel (*C* and *D*) shows the same data on a logarithmic scale. In panel *C*, blue and red dashed lines represent pre-specified levels of partial and complete B-cell depletion, respectively. Parametric ANOVA was performed comparing different timepoints against baseline. Significance was corrected for multiple testing. *n* = 24 patients (6/dose). **P < 0.01, ***P < 0.001, ****P < 0.0001.

Given as a single dose, rituximab was effective at acutely depleting B cells (*Figure 2*). For all doses, rituximab caused a mean 96.3% [95% confidence interval (CI) 93.8–98.8%] depletion of the B-cell count (*Figure 2*) within 30 min of starting the infusion. This was significantly lower than baseline for all doses (*Figure 2B*, 200 mg *P* = 0.0004, 500 mg *P* < 0.0001, 700 mg *P* = 0.0001, 1000 mg *P* < 0.0001). At 6 h, a rebound in B-cell numbers was seen for the 200, 500, and 700 mg doses, which appeared dose-dependent (Supplementary material online, *Figure S1A*), whilst for the 1000 mg dose, B cells remained low and did not rebound. On Days 6 and 14, B-cell counts were significantly lower than baseline for all doses (*Figure 2*). At 6 months, recovery of total B-cell count appeared dose-dependent (mean % recovery = 57.8%, 20.8%, 22.6%, and 3.9% for 200, 500, 700, and 1000 mg doses, respectively; *Figure 2*) with the 200 mg dose not significantly different compared to baseline (*P* = 0.63). When comparing between doses, a difference between B-cell count was significant at 6 months only between the 200 mg and 1000 mg dose (*P* = 0.02).

**Figure 3:**
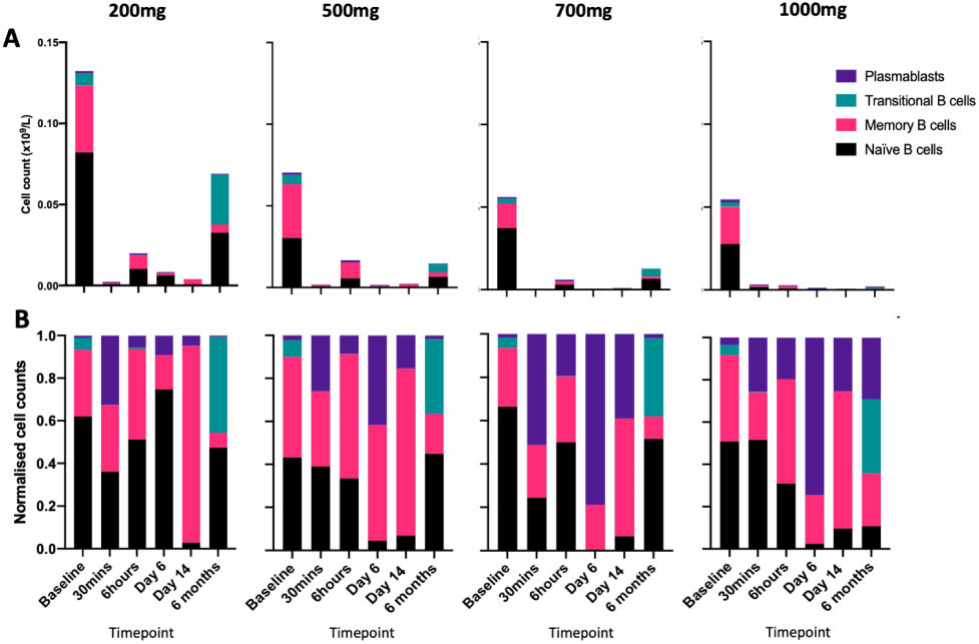
Parts of whole stacked bar graph of B-cell subsets. The four columns of graphs show the data from the four rituximab doses (200, 500, 700, and 1000 mg). Panel *A* shows the absolute counts of B-cell subsets as a ‘part of whole’ stacked bar graph. Each colour represents a B-cell subset. Plasmablasts numbers are low and therefore poorly visible. The total of all the subsets in each bar equals total B-cell count. Panel *B* shows the same data but normalized so each subset is represented as a percentage of the whole. *N* = 24 patients (6/dose).

Rituximab depleted all B-cell subsets (*Figure 3* and Supplementary material online, *Figure S1*). At 6 months, naïve, transitional, and memory B cells appeared to repopulate in a dose-dependent manner with lower numbers seen at the higher doses. The overall result was a lower proportion of memory B cells and higher proportion of transitional and naïve B cells at 6 months compared to baseline (*Figure 3*).

### 3.3 Effect of rituximab on immune cells

As an exploratory outcome, we examined the effect of rituximab on other immune cells (Supplementary material online, *Figures S2*–*S4*). For the 1000 mg dose, lymphocyte count and percentage appeared to decrease 30 min after starting the rituximab infusion, with further reductions seen at 6 h (Supplementary material online, *Figure S2*). After this, levels appeared to return back to pre-dosing levels by Day 6. The dynamic change in lymphocyte count was comprised not only of changes in B-cell numbers but also of CD4^+^ and CD8^+^ T cells (Supplementary material online, *Figure S4*). Monocyte percentage and count showed the same pattern (Supplementary material online, *Figure S2E* and *F*), whilst the opposite was seen for neutrophils with increases seen at 30 min and 6 h and a return back to baseline by Day 6 (Supplementary material online, *Figure S2G* and *H*). No changes were seen in eosinophils, basophils or regulatory T cells.

### 3.4 Effect of rituximab on biomarkers and immunoglobulins

**Figure 4:**
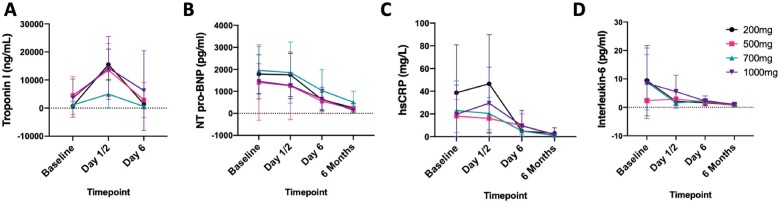
Grouped data showing cardiac biomarkers. Each panel shows the mean and 95% CI for each of the four rituximab doses (200, 500, 700, and 1000 mg) at different timepoints. Panels *A*–*D* show troponin, NT pro-BNP, hsCRP, and interleukin-6, respectively. *N* = 24 patients (6/dose). BNP, NT-pro-B-type Natriuretic Peptide; hsCRP, high-sensitivity C-reactive protein.

Troponin I and high-sensitivity CRP (hsCRP) levels peaked at Day 1 and decreased by Day 6 and 6 months (*Figure 4A and C*). NT-pro-BNP and IL-6 levels decreased from baseline with the lowest values seen at 6 months (*Figure 4B and D*). Lipids profiles were measured at baseline and 6 months with decreases seen in total cholesterol, LDL-C, and non-HDL. No changes were seen in HDL-C and triglycerides (Supplementary material online, *Figure S5*).

**Figure 5:**
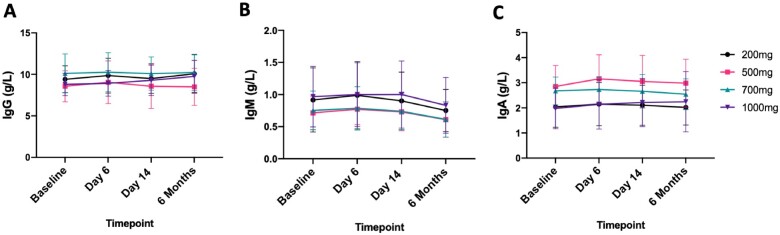
Grouped data showing immunoglobulin counts. Each panel shows the mean and 95% CI for each of the four rituximab doses (200, 500, 700, and 1000 mg) at different timepoints. Panels *A*–*C* show IgG, IgM, and IgA, respectively. *N* = 24 patients (6/dose). Ig, immunoglobulins.

Rituximab had no effect on either total IgG (*P* = 0.54, 0.054), IgM (*P* = 0.58, *post hoc* non-significant) and IgA (*P* = 0.2, 0.085) levels (*P*-values for dose and timepoint interaction, respectively) (*Figure 5*), or on levels of IgM and IgG against OSEs (Supplementary material online, *Figure S6*).

The RITA-MI trial did not mandate echocardiography for patients. However, as is standard clinical practice, 12/24 patients had an echo on index admission with a follow-up echo within 6 months. These patients were not selected by the research team. There was a trend towards improved LV ejection function at follow-up [mean at index and follow-up: 46.5 vs. 54.3%; mean increase in ejection fraction of 7.8% (95% CI 3.11–12.6)] (Supplementary material online, *Figure S7*).

## 4. Discussion

In this trial, we report the first safety experience of rituximab in patients with acute MI. Overall, we found a favourable safety profile without any dose effect for AEs, which was confirmed by an independent DSMB. Moreover, we report that a single infusion of rituximab profoundly depletes circulating mature B cells as early as 30 min following the start of the infusion and at doses as low as 200 mg, suggesting the feasibility of a ‘fire and forget’ approach, with rapid and sustained modulation of immune responses during the few critical months following acute MI.

Rituximab use in patients with haematological cancers and autoimmune disease has been associated with both atrial and ventricular arrhythmias,^17–19^ and acute myocardial ischaemia.^20^ Given that patients immediately post-STEMI have evolving cardiac scar and inherent electrophysiological instability, it was reassuring that throughout >150 h of continuous cardiac monitoring during and after dosing, no new arrhythmias were detected. This may be related to the high rate of beta-blocker use in the trial. Furthermore, it did not appear that rituximab caused any cardiac ischaemia. In our study, the incidence of infusion-related reactions was 12.5%, which is lower than the reported incidence of 30–35% in oncology or autoimmune patients. Infection incidence was comparable to observation data on general patients with MI,^21^ with four patients reporting infections in the 6 months follow-up period. However, by design our study population is likely younger and ‘healthier’ than the overall MI population.

The most common groups of AEs were gastrointestinal disturbances, which included diarrhoea, indigestion, and abdominal pain. This may be related to the fact that the majority of patients did not have a previous cardiac history and, as a result, were started naïve on the standard range of secondary prevention medication. This likely triggered the gastrointestinal problems they experienced which settled without intervention and on follow-up. The increased liver function tests were likely related to the high-dose statin therapy (atorvastatin 80 mg daily) which is a known side effect.^22^ These patients were asymptomatic, and abnormal tests results resolved on follow-up without intervention.

Rituximab has been used successfully in a case series of patients with inflammatory cardiomyopathy^23^ and also been shown to improve endothelial function,^24^ arterial stiffness, and decrease carotid intima-media thickness in patients with rheumatoid arthritis.^25^^,^^26^ However, unlike those chronic states, a rapid depletion of circulating B cells need to be achieved for rituximab to be potentially useful in patients with acute STEMI. To the best of our knowledge, this is the first time the effect of rituximab has been studied in the hyper-acute 30 min timepoint, and where a robust and effective depletion of circulating B cells is documented. Indeed, in all patients except one, partial depletion was achieved (i.e. level of total B cells below 0.005 ×10^9^/L from a mean baseline level of 0.08 ×10^9^/L) within 30 min of the infusion starting. At this timepoint, only 25 mg of rituximab would have been infused into the patients. This finding is important as we are targeting the initial inflammatory phase of damage seen in acute MI. Acute B-cell depletion is expected to limit the number of inflammatory cytokines produced by activated B cells secondary to ischaemic injury and would contribute to a rapid de-activation and limitation of the inflammatory response post-MI. The first few weeks to months following an acute MI probably constitute the most critical phase of inflammation-dependent cardiac remodelling. It was therefore important to ensure that the various doses of rituximab used in this study were able to induce a sustained B-cell depletion. B cells are known to reconstitute a few months after a single rituximab infusion. Interestingly, the composition of the reconstituted B cells at 6 months showed lower numbers of memory B cells and higher number of naïve and transitional B cells compared to baseline (*Figure 3*). We expect this profile of reconstituted B cells to still be compatible with a sustained regulation of the immune response given the reparative and regulatory phenotype of transitional B cells.^27^

Another novel finding is the profound acute changes to the leucocyte population. The decrease in lymphocyte count is comprised of decreases in both B- and T-cell populations. The mechanism is unclear. However, we can hypothesis that T cells were sequestered into secondary lymphoid organs as B cells are removed from these structures and then returned back to the circulation slowly after days. Monocytes have been shown to be important for B-cell depletion in mouse models^28^^,^^29^ where compromised blood flow through the liver affected anti-CD20 antibody efficacy whilst splenectomy had minimal effect.^28^^,^^30^ Therefore, the acute decrease seen may be related to trafficking of monocytes into various tissues like the liver, which then take part in the antibody-dependent phagocytosis of B cells, and not consistent with the natural history of MI. We see an acute and transient increase in neutrophils. However, it is not possible in this study to evaluate if this was related to rituximab treatment or to the effects of methylprednisolone.

One of the concerns with rituximab therapy was a possible decrease in IgG which has been shown in patients with rheumatoid arthritis to predict risk of infection.^31^ This was not observed. Furthermore, IgM to OSEs were not affected, but we do not have a control group and therefore cannot compare the natural history of IgM to OSEs post-MI over a 6-month time period to what was observed. Most IgM to OSEs are suggested to be produced by B1 cells and potentially atheroprotective.^11^^,^^32^^,^^33^ Whether these results show that rituximab does not deplete B1 cells is difficult to ascertain. Although human B1 cells express CD20, they are not frequently found in blood. Moreover, tissue ‘resident’ populations of B cells, like peritoneal B1 cells, have been shown to be more resistant to depletion with anti-CD20, including those that express CD20 (e.g. germinal centre B cells and marginal zone B cells).^30^^,^^34^ With regard to cardiovascular biomarkers, the trends in troponin, NT-pro-BNP, IL-6, hsCRP, and lipid profile are all consistent with the natural history of MI and high dose atorvastatin therapy. No safety issues were raised by these biomarkers.

Our study has some limitations. It was a small single-centre study with a limited number of patients and therefore we are still learning the safety of rituximab in MI. A lack of a control group makes interpretation of some of the results challenging given the dynamic immune environment after an MI. Furthermore, the majority of our patients are white males, which limits the generalizability of this data to other populations. We aim to address this in our phase 2b study, which is a multicentre, international study across eight countries. In this trial, methylprednisolone was used as a pre-mediation to prevent infusion-related reaction as is standard clinical practice. Various steroids have been tested in MI in the early 1970s/1980s in small clinical trials with mixed results, which was balanced with potential risks, including post-MI myocardial wall thinning.^35^ It is also important to consider that all the trials used much higher doses of steroids. For example, in the largest prospective randomized controlled trial, the total dose was 6 g of intravenous hydrocortisone.^36^ Therefore, it is unlikely that our dose of 100 mg of methylprednisolone had any meaningful myocardial effect.

The difference in B-cell depletion between the 1000 mg and 200 mg dose is only obvious at 6 months where the highest dose caused prolonged depletion with a lower percentage of recovery. The question of ideal length of B-cell depletion in patients with MI is unclear. Furthermore, in patients with MI, the temporal changes in the cardiac immune response are poorly understood. Therefore, both safety and ideal dose will need further investigation in a phase 2b trial. The latter, called RITA-MI 2 and funded by the European Commission, will be a multinational randomized double-blind placebo-controlled clinical trial to assess the impact of B-cell depletion using rituximab (200 mg and 1000 mg) on LV dysfunction and cardiac remodelling after acute MI.

## 5. Conclusion

In this first RITA-MI trial, we have shown promising safety and feasibility data for the use of rituximab in patients with STEMI. In addition, novel immunological insights have been gained into its effects in patients. This work informs the use of rituximab in larger phase 2b clinical trials.

## Supplementary material

Supplementary material is available at *Cardiovascular Research* online.

## Authors’ contributions

Z.M. conceived the idea. T.X.Z., S.P.H., and Z.M. designed the trial. T.X.Z., M.A.U.-R., S.V., R.K., and S.F. performed the study. S.P.H. was the principal investigator. A.P.S., T.X.Z., and C.J.B. contributed to the lab data. T.X.Z., Y.-D.C., and M.M. analysed the data. H.A.-O. contributed intellectually and revised the paper. T.X.Z., S.P.H., and Z.M. wrote the manuscript and all the authors approved the final version.

## Supplementary Material

cvab113_Supplementary_Data

## Notes

Translational perspective Selective depletion of B cells using anti-CD20 antibody has been shown to be beneficial in various pre-clinical models of myocardial infarction with additional supportive clinical observational data. Our research provides the first translational step in using this strategy in patients. We found rituximab to be safe and effective in rapidly and profoundly depleting B cells in patients after an acute myocardial infarction. This work has directly led to the funding and setup of a multicentre, international, randomized, double-blind, placebo-controlled, phase 2b clinical trial which should give us an indication of this strategy’s clinical efficacy.
