# Supplementary material for: Rituximab in patients with acute ST-elevation myocardial infarction: an experimental medicine safety study
Source: Cardiovasc Res. 2021 Mar 30;118(3):872–82. doi: 10.1093/cvr/cvab113 (PMC8859640; doi:10.1093/cvr/cvab113)
Supplement: cvab113_Supplementary_Data [file cvab113_supplementary_data.pdf]

# SUPPLEMENTARY MATERIAL

**Rituximab in Patients with Acute ST-elevation Myocardial Infarction (RITA-MI): an Experimental Medicine Safety Study**

Rituximab in acute STEMI: a phase 1/2a study

Tian X. Zhao[1], Muhammad Aetesam-Ur-Rahman[2], Andrew P. Sage[1], Saji Victor[3], Rincy Kurian[3], Sarah Fielding[4], Hafid Ait-Oufella[5], Yi-Da Chiu[4], Christoph J. Binder[5], Mikel Mckie[4], Stephen P. Hoole[2], Ziad Mallat[1,6]

CVR-2021-0147

| Dose Level (mg) | 200 | 500 | 700 | 1000 |
|---|---|---|---|---|
| **Total number of AEs** | 18 | 19 | 10 | 17 |
| **Category** | | | | |
| Allergic dermatitis | 1 (5.6) | 0 (0.0) | 0 (0.0) | 0 (0.0) |
| Arrhythmia | 0 (0.0) | 1 (5.3) | 2 (20.0) | 1 (5.9) |
| Breathlesness | 0 (0.0) | 1 (5.3) | 0 (0.0) | 0 (0.0) |
| Bruising | 0 (0.0) | 0 (0.0) | 0 (0.0) | 1 (5.9) |
| Chest pain | 1 (5.6) | 3 (15.8) | 1 (10.0) | 0 (0.0) |
| Cystitis | 0 (0.0) | 0 (0.0) | 0 (0.0) | 1 (5.9) |
| Delayed discharge and further PCI | 0 (0.0) | 1 (5.3) | 0 (0.0) | 0 (0.0) |
| Dry cough | 1 (5.6) | 0 (0.0) | 0 (0.0) | 1 (5.9) |
| Elective procedure | 1 (5.6) | 0 (0.0) | 0 (0.0) | 1 (5.9) |
| Elevated Liver function tests (LFTs) | 2 (11.1) | 1 (5.3) | 0 (0.0) | 2 (11.8) |
| Epistaxis | 0 (0.0) | 3 (15.8) | 0 (0.0) | 1 (5.9) |
| Exertional Breathlessness | 0 (0.0) | 0 (0.0) | 0 (0.0) | 1 (5.9) |
| Face numbness- transient ischemic attack | 0 (0.0) | 0 (0.0) | 0 (0.0) | 1 (5.9) |
| Fresh PR Bleeding | 0 (0.0) | 1 (5.3) | 0 (0.0) | 0 (0.0) |
| GI disturbance | 5 (27.8) | 5 (26.3) | 2 (20.0) | 4 (23.5) |
| Hand numbness- transient ischemic attack | 0 (0.0) | 0 (0.0) | 0 (0.0) | 1 (5.9) |
| Hyponatraemia | 0 (0.0) | 0 (0.0) | 1 (10.0) | 0 (0.0) |
| Infection | 1 (5.6) | 1 (5.3) | 1 (10.0) | 1 (5.9) |
| Infusion related reaction | 3 (16.7) | 0 (0.0) | 0 (0.0) | 0 (0.0) |
| Insomina | 1 (5.6) | 0 (0.0) | 0 (0.0) | 0 (0.0) |
| Joint Pain - rheumatoid arthritis | 0 (0.0) | 0 (0.0) | 0 (0.0) | 1 (5.9) |
| Lethargy | 0 (0.0) | 1 (5.3) | 0 (0.0) | 0 (0.0) |
| Musculoskeletal injury | 1 (5.6) | 0 (0.0) | 1 (10.0) | 0 (0.0) |
| Palpitations | 1 (5.6) | 0 (0.0) | 1 (10.0) | 0 (0.0) |
| Renal impairment | 0 (0.0) | 1 (5.3) | 0 (0.0) | 0 (0.0) |
| Sinusitis | 0 (0.0) | 0 (0.0) | 1 (10.0) | 0 (0.0) |

**Supplementary Table 1.** All adverse events (AEs) stratified by dose group. First number is number of AEs whilst number in brackets is percentage.

**Supplementary Table 2. Details of 5 serious adverse events:**

1. Patient 112 (group 2) had uneventful dosing of rituximab and was discharged from the ward on day 4 post-MI when he developed chest pain in the discharge lounge. The pain lasted for a few minutes and he was reviewed by the clinical team and admitted back to the ward. He did not have any new changes on ECGs and troponin was continuing on the downward trend. Later that day, he was taken back to the lab. Coronary angiogram showed his LAD stent from the index procedure was patent. He had a non-culprit OM1 lesion, which was initially left at the time of index procedure and was then stented without complication during the repeat procedure. On review of index and repeat angiogram by the PI, it was felt that the OM1 lesion was unchanged. Therefore, the SAE was assessed as not related to rituximab. SAE Category: Admission to hospital
2. Patient 112 had an episode of angina 3months after index event, which started at home. The pain persisted so he went to Ipswich hospital. His angina settled in A&E however he was admitted for monitoring. There were no ECG changes, and troponin was negative. He was discharged the next day with the addition of Nicorandil 10mg. No other procedures were performed. Due to the temporal disassociation and significant residual coronary disease, the SAE was assessed as unrelated to the IMP. SAE Category: Admission to hospital.
3. Patient 114 (group 2) had a previous history of coronary artery bypass surgery for angina. Admitted for anterior STEMI and was successfully dosed in the trial without complication. Echo during index admission showed severe LV impairment and was discharged on day 3 post-MI without any symptoms. Two weeks after his index MI, he was admitted to Norfolk and Norwich hospital with palpitations. He was diagnosed with ventricular tachycardia (VT) and treated with DC cardioversion in A&E. He was admitted to the ward and repeat ECHO showed an EF of 30%. The diagnosis was VT secondary to ischaemic cardiomyopathy. He was managed with an implantable cardiac defibrillator (ICD) and discharged home without any further complications. Since the patient had severe LV impairment at the index admission before dosing, which was the likely cause of his VT, the SAE was assessed as unrelated to the IMP. SAE Category: Admission to hospital
4. Patient 116 (group 2) was admitted to Peterborough General Hospital from home 9 days after index admission, with pain in the epigastric and chest area. No ECG changes were found, and troponin blood tests were negative. Admitted to complete investigations and for observation. A diagnosis of indigestion was made, and the patient was discharged the day after. Subsequent outpatient upper GI endoscopy demonstrated hiatus hernia. The SAE was assessed as unrelated to the IMP. SAE Category: Admission to hospital
5. Patient 306 (group 3) had a known history of chronic rate-controlled atrial fibrillation (AF). Before and during IMP dosing, the patient’s heart rate was 80-90bpm in AF. On Day 1 post dosing, the patient's heart rate increased to 100-120bpm. The patient did not have any symptoms. This was 17hours after the infusion had finished. He was seen by the clinical team who started him on oral digoxin. They decided to keep him another day for monitoring of his heart rate. The next day the patient’s heart rate had resolved back to normal and he was discharged. Since the change in AF rate occurred 17hours after the completion of rituximab infusion, it was assessed that the SAE was not related to IMP. SAE Category: Prolongation of hospital stay.

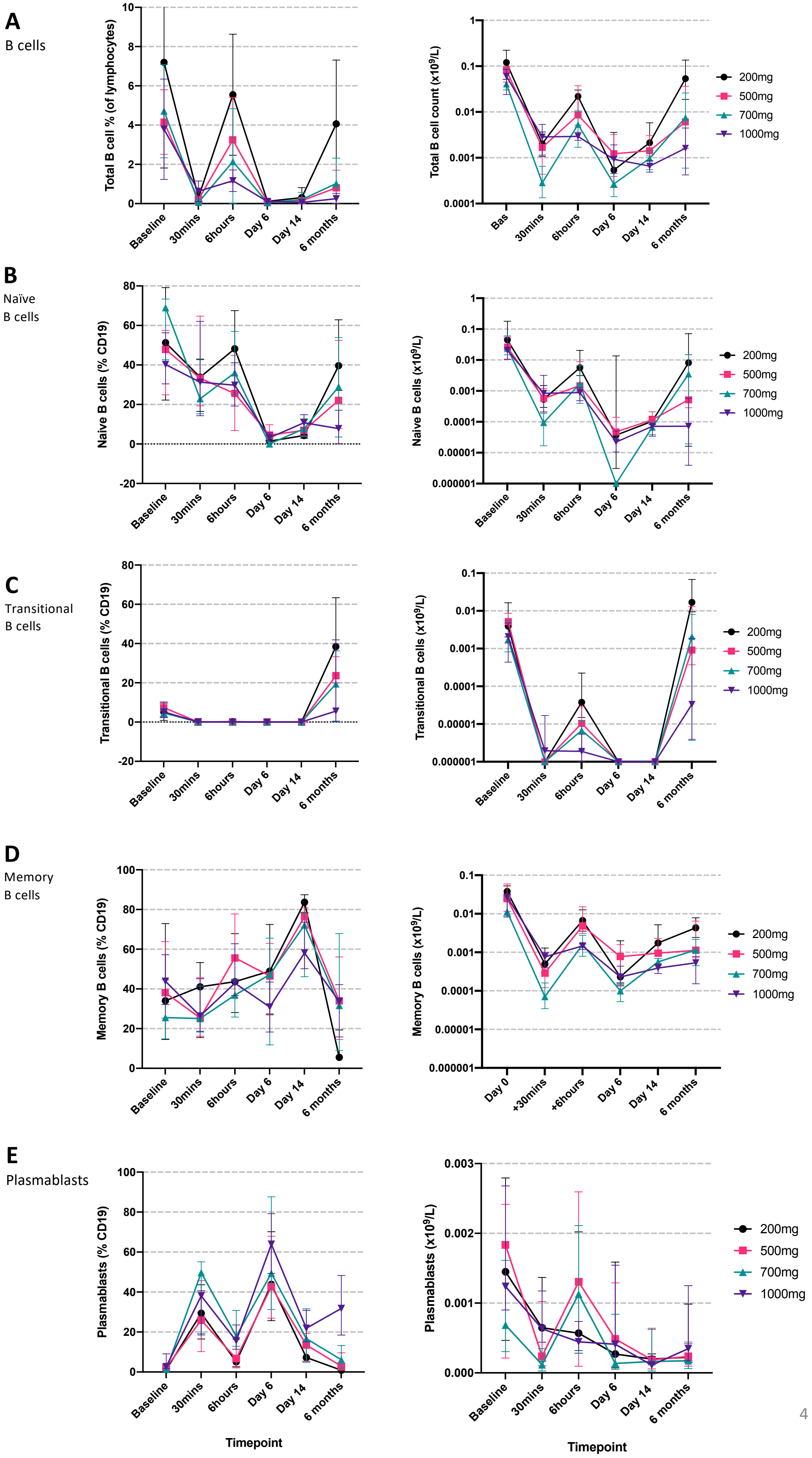

A
B cells
Total B cell % (of lymphocytes)
Total B cell count (x10^9/L)
B
Naïve
B cells
Naive B cells (% CD19)
Naive B cells (x10^9/L)
C
Transitional
B cells
Transitional B cells (% CD19)
Transitional B cells (x10^9/L)
D
Memory
B cells
Memory B cells (% CD19)
Memory B cells (x10^9/L)
E
Plasmablasts
Plasmablasts (% CD19)
Plasmablasts (x10^9/L)
Baseline
Bas
Day 0
30mins
+30mins
6hours
+6hours
Day 6
Day 14
6 months
Timepoint
200mg
500mg
700mg
1000mg

**Supplementary Figure 1. Comparison between groups for B cells and subsets**. Each panel shows the grouped data from the 4 rituximab doses (200, 500, 700, and 1000mg). Panels on the left show % of cells (of either lymphocytes or CD19+ B cells).Panels on the right show absolute counts.  Points and error bars represent median and IQR. Panel **A** shows B cells, whilst panels **B** – **E** show B cell subsets. n=24 patients (6/dose group).

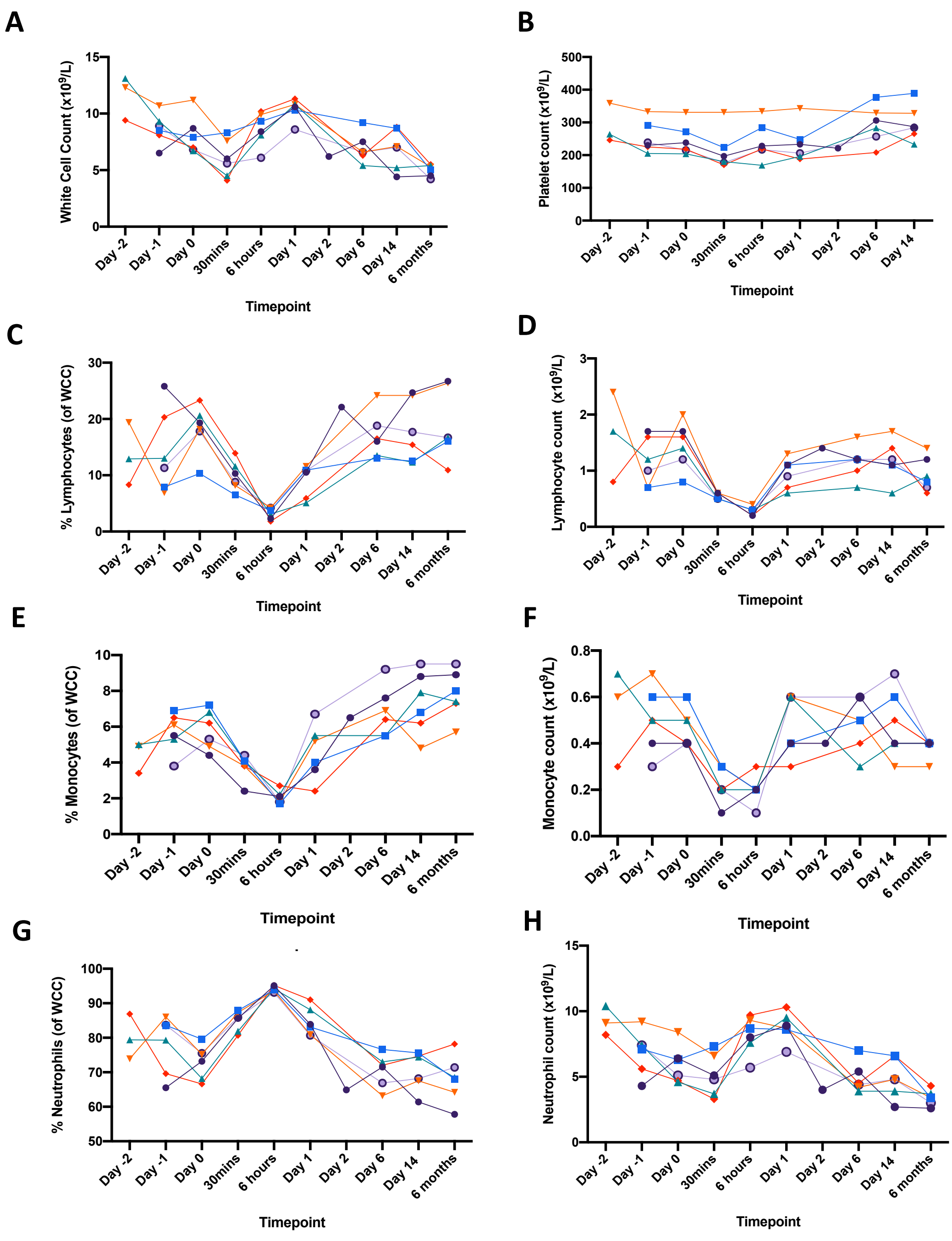


**Supplementary Figure 2. The effect of 1000mg of rituximab on the clinical full blood count**. Patient level data. Each dot and line represents a single patient. 30 mins and 6 hours represent time after the start of the rituximab infusion. n=6 patients.

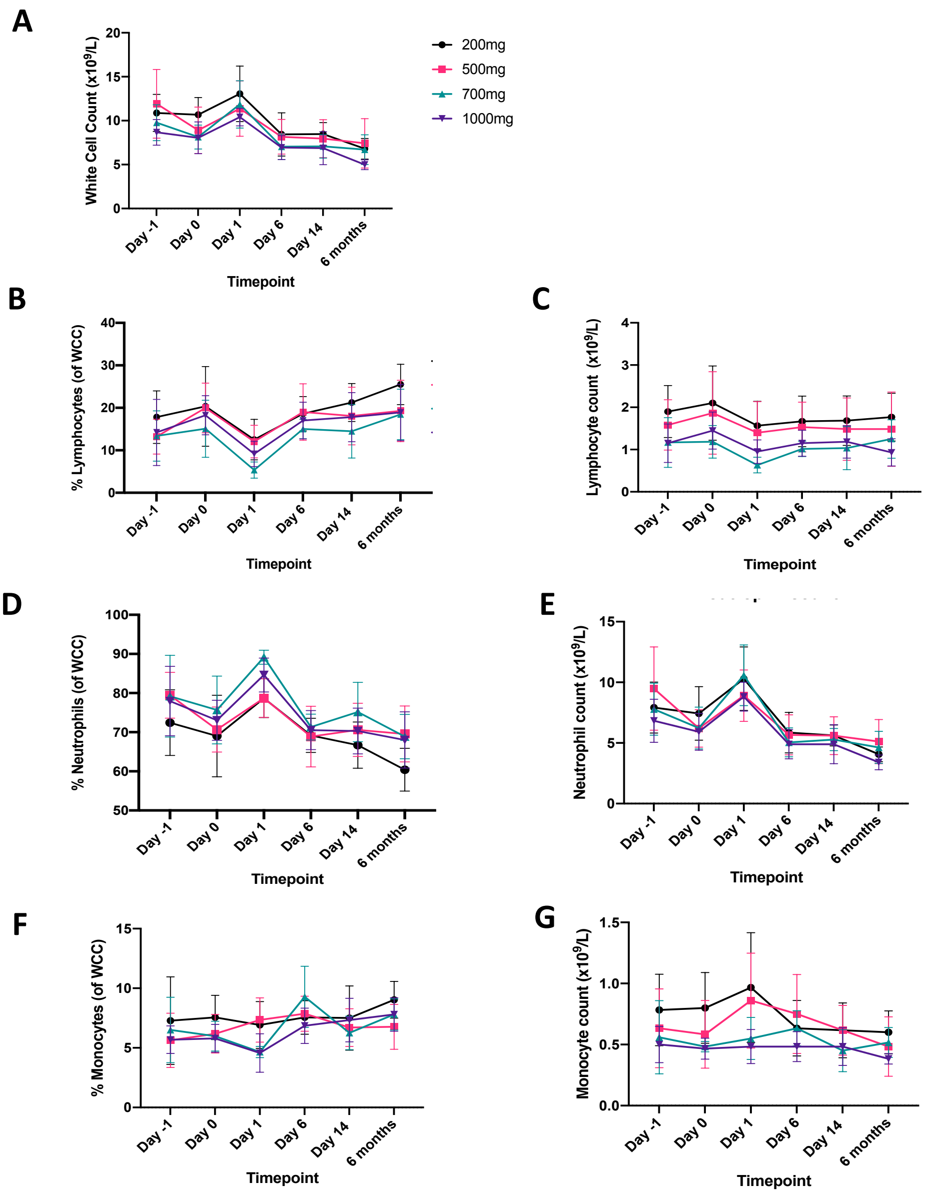


**Supplementary Figure 3. Grouped data showing full blood count results**. Each panel shows the mean and 95% CI for each of the 4 rituximab doses (200, 500, 700, and 1000mg) at different timepoints. Panels **A** shows the white cell count (WCC). Below that, panels on the left show various cell counts as % of WCC, and panels on the right show absolute counts. n=24 patients (6/dose group).

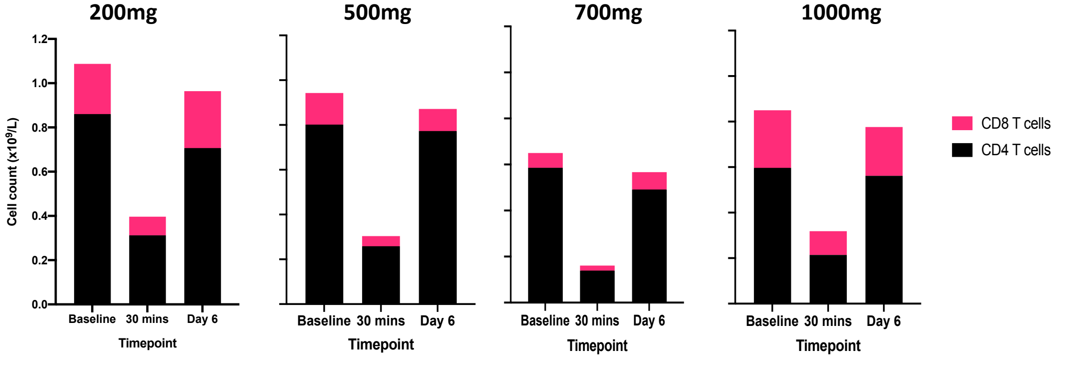


**Supplementary Figure 4. Parts of whole stacked bar graph of T cells subsets**. Each panel shows the data from each of the 4 rituximab doses (200, 500, 700, and 1000mg). Each bar chart represents CD4+ and CD8+ T cell counts. n=24 patients (6/dose group).

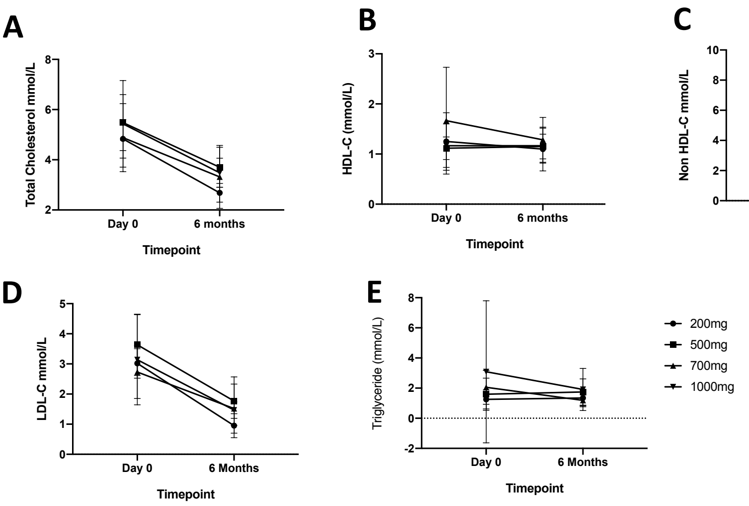


**Supplementary Figure 5. Grouped data showing lipid profile**. Each panel shows the mean and 95% CI for each of the 4 rituximab doses (200, 500, 700, and 1000mg) at different timepoints. n=24 patients (6/dose group).

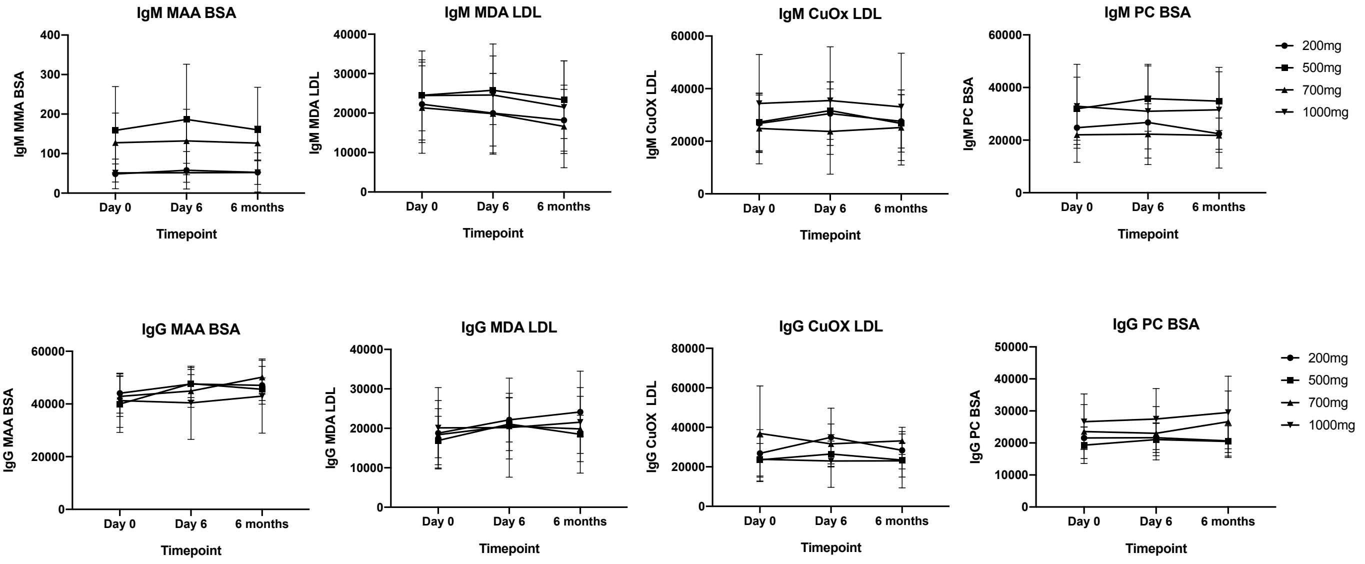


**Supplementary Figure 6. Grouped data showing immunoglobulins for oxidation-specific epitopes (OSEs)**. Each panel shows the mean and 95% CI for each of the 4 rituximab doses (200, 500, 700, and 1000mg) at different timepoints. Ig = immunoglobulins, BSA = bovine serum albumin, MDA = malondialdehyde-modified, MAA = maldondialdehyde -acetaldehyde, LDL = low-density lipoproteins, Cu-OxLDL =: copper-oxidized LDL, PC = phosphorylcholine. n=24 patients (6/dose group).

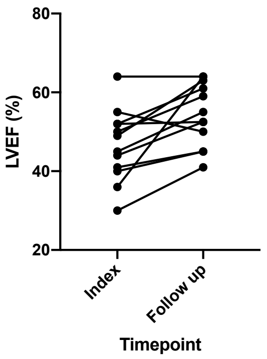


**Supplementary Figure 7. Echocardiogram data.** Paired data with each line representing a single patient and their two echocardiogram results. n=12 patients. LVEF = left ventricular ejection fraction

# Supplementary methods

## Lymphocyte FACS analysis

Lymphocyte subset analysis was performed at the NIHR Cambridge BRC Cell Phenotyping Hub. Samples were analysed within 4 hours of collection in BD Vacutainer CPT Mononuclear Cell Preparation Tube with sodium heparin. Samples were then processed to isolate PBMCs. There were then stained for B cells and subsets using: anti-CD3 (e605-laballed), anti-CD19 (v450-labelled), anti-CD20 (PAC-eF780-labelled), anti-CD38 (APC AF647-labelled), anti-CD27 (PE7-labelled), anti-CD24 (PerCP-Cy5.5-labelled), anti-IgG (PE-labelled), and anti-IgD (FITC-labelled).

B cell subsets were defined as the following, naive B cells: CD19+ CD27- IgD+. Transitional B cells: $CD19^{+}$ $CD27^{-}$ $IgD^{+}$ $CD24^{hi}CD38^{hi}$, total memory B cells equalled combined switched ($CD19^{+}CD27^{+}IgD^{-}$) and unswitched memory ($CD19^{+}CD27^{+}IgD^{+}$) B cells. Plasmablasts: $CD19^{+}IgD^{-}CD27^{hi}CD38^{hi}$

T cells and subsets were stained using anti-CD3 (e605-laballed), anti-CD4 (APC AF647-labelled), anti-CD8 (e655-labelled), anti-HLA-DR (v450-labelled), anti-CD38 (APC AF647-labelled), anti-CCR6 (PE7-labelled), anti-CD45RA (PerCP-Cy5.5-labelled), anti-CCR7 (PE-labelled), and anti-CXCR3 (FITC-labelled) antibodies.

Regulatory T cells were stained using anti-CD3 (e605-laballed), anti-CD4 (APC AF647-labelled), anti-CD25 (PE-labelled), anti-CD127 (APC AF647-labelled) antibodies. Treg cells were defined as $CD3^{+}CD4^{+}CD25^{+}CD127^{low}$.

## Biomarker analysis

Biomarkers were assessed using banked serum at the Core Biochemical Assay Laboratory, Cambridge. It was analysed in duplicate using ultra-sensitive electrochemical luminescence immunoassay on the Mesoscale Discovery assay platform and read on the MesoScale Diagnostics Sector Imager 6000. All reagents and calibrators were supplied by MesoScale Discovery.

## Safety bloods comprised of:

Haematology (Haemoglobin (Hb), White Blood Count (WBC), Platelet Count (Plt), Red Cell Count (RBC), Haematocrit (HCT), Mean Cell Volume - Red cell (MCV), Mean Cell Haemoglobin (MCH), Differential White Cell Count (Neutrophils, Lymphocytes, Monocytes. Eosinophils, Basophils)); Clinical biochemistry (Sodium, potassium, urea, creatinine, liver function (ALT, ALP, albumin, bilirubin), calcium.
